# Reducing spatial variability of soybean response to rhizobia inoculants in farms of variable soil fertility in Siaya County of western Kenya

**DOI:** 10.1016/j.agee.2018.01.007

**Published:** 2018-07-01

**Authors:** M. Thuita, Bernard Vanlauwe, E. Mutegi, C. Masso

**Affiliations:** International Institute of Tropical Agriculture, PO Box 30772-00100, Nairobi, Kenya

**Keywords:** Nodule occupancy, Biological nitrogen fixation, Soybean inoculants, Soil fertility gradient

## Abstract

Soybean grain yields in sub-Saharan Africa have remained at approximately 50% below those attained in South America despite numerous efforts. A study was conducted in Siaya County (western Kenya) involving 107 farms with soils of different fertility status. The main objective was to test combinations of two inoculants (Legumefix and Biofix) and nutrient sources (Minjingu and Sympal) to raise soybean grain yields. Inoculation used Legumefix or Biofix with and without Minjingu or Sympal in a factorial design. There was soil acidity and a widespread deficiency of potassium, nitrogen, and phosphorus. Inoculation and nutrient source led to increases in nodulation and nodule occupancy. For grain yields the response varied from nil in some sites to high increases in others. Highest grain yields (3000–4000 kg ha^−1^) were obtained with Legumefix + Sympal (12% of the farmers testing it). The formulation of the nutrient source was important to meet other nutrient deficiencies in most of the soils. Farmers using Legumefix + Sympal require yield increases of 35% for profitability (Value cost ratio of 3) while farmers using Minjingu + inoculant require a yield increase of at least 68%. Inoculants used alone were most profitable but this is advisable only when farmers are too resource constrained to afford fertilizer. For sustainable yields Legumefix + Sympal or Biofix + Sympal were recommended.

## Highlights

• Soybean yields in SSA are low and variable compared to North and South America.
• Low soil fertility and poor quality rhizobia inoculants contribute to low yields.
• Integrated soil fertility management (ISFM) packages for soybean to reduce yield variability.
• Inoculation with Rhizobia and addition of Sympal gave yield of up to 4 t ha^−1^.
• Yield increase of 35–70% required for profitability of ISFM packages.

## 1 Introductions

Soybean grain yields in most of sub-Saharan Africa (SSA) remain relatively low compared to those achieved in South America and USA (Mpepereki et al., 2000). In other regions, annual average yield increases have been reported of 31 kg ha^−1^ in the United States (Specht et al., 1999) and 28 kg ha^−1^ worldwide (Wilcox, 2004). To achieve their high yield potential, soybean must sustain high rates of photosynthesis and accumulate large amounts of nitrogen (N) in seeds (Salvagiotti et al., 2008). Nitrogen exists in leaves primarily as ribulose biphosphate carboxylase/oxygenase and there is generally a strong relationship between N per unit leaf area and photosynthesis (Sinclair, 2004). Soil is the main source for most plants but N remains a major plant growth limiting nutrient in SSA (Sanchez et al., 1997). The alternative source for soybean is biological nitrogen fixation (BNF) through symbiosis with rhizobia. Worldwide some 44–66 million tonnes (t) of N_2_ are fixed annually by agriculturally important legumes with another 3–5 million t fixed by legumes in natural ecosystems, providing nearly half of all N used in agriculture (Smil, 1999; Graham and Vance, 2000). The contribution by BNF could be increased by improving the nutrition of legumes, attending to edaphic constraints such as soil acidity and drought, and breeding varieties that’s target the symbioses with rhizobia (Graham and Vance, 2000).

Rhizobia inoculants have proven to be a more viable and sustainable approach to meet the its high N demand estimated at 80 kg N per 1000 kg of soybean grain (Hungria et al., 2006). Soybean often requires inoculation when introduced in new environments including SSA. Owing to challenges associated with inoculum production, handling, and storage, breeding for promiscuity was proposed (Kueneman et al., 1984). However, the use of promiscuous soybean did not yield the expected outcome as indigenous rhizobia are often not present in high enough numbers and nitrogen fixation efficiency is relatively low (Musiyiwa et al., 2005; Zengeni and Giller, 2007). Inoculation has been proposed and shown to be beneficial even on promiscuous varieties (Thuita et al., 2012).

In their review, Divito and Sadras (2014) demonstrated that nutrients such as potassium (K) and sulfur (S) play a major role in nodule function and BNF. This is in addition to the widely documented role of phosphorus (P) (Almeida et al., 2000; Olivera et al., 2004; Schulze, 2006). Sulieman et al. (2013) reported that legumes relying on BNF generally require more P, K, and S than those that do not. Even though in most of SSA, N and P are documented as the most limiting nutrients and most fertilizer inputs mainly contain them it is possible that limitations in other nutrients may be the reason why yields of soybean have remained relatively low with averages of about 2 t ha^−1^ (Mpepereki et al., 2000). Addressing these other possible constraints could be the key to increasing yields to a level nearer to the averages obtained in South America (Hungria et al., 2006). Approaches by way of integrated soil fertility management (ISFM) (use of fertilizer + organic inputs, improved varieties, and rehabilitation of non-responsive soils) have been proposed as a viable sustainable, and environmentally friendly approach (Vanlauwe et al., 2010) and must be used to increase agricultural productivity (Okalebo, 2009; Garnett et al., 2013). This study aimed at testing two ISFM components (2 inoculants and 2 nutrient sources) to reduce the spatial variability of soybean yields and the yield gap.

## 2 Material and methods

### 2.1 Field trial

The trials were conducted in five regions (Boro, Ugunja, Ukwala, Wagai, and Yala) of Siaya County in western Kenya. Siaya County is located at 00 08.468′N, 34°25.378′E, and 1336 m asl. The experimental sites were in the lower midland 1 (LM1), lower midland 2 (LM2), and upper midland 1 (UM1). These agroecological zones experience bimodal rainfall with long rains (LR) from March to July and short rains (SR) from late August to December (Jaetzold et al., 2007). Average annual rainfall is 1500 mm (GOK, 1997). The soils are mainly Ferralsols and Acrisols in the higher (hilly and elevated) areas and Vertisols in the lower areas (near river valleys and plains).

The farmers who participated in the trials were selected randomly First all the villages in each region were listed and random numbers were generated using Microsoft excel. From each selected village the names of all the farmers in the village were obtained and four were selected using random numbers. In total, 107 randomly selected farmers participated in the trials. Initial soil samples were taken for analysis before treatments were applied. Available P was determined using the Mehlich 3 method (Mehlich, 1984); pH (H_2_O) was determined as described by Okalebo et al. (2002). Exchangeable K, Ca, Mg, Total N (%), and organic carbon (%) were determined as described by Tekaligu et al. (1991) and Anderson and Ingram (1993).

### 2.2 Treatment structure and application

Two soybean inoculants were tested. Legumefix^®^ soya from Legume technology (UK) containing *Bradyrhizobium japonicum* strain 532c (Thuita et al., 2012) and Biofix^®^ soya from MEA Ltd (Kenya) containing *Bradyrhizobium diazoefficiens* strain USDA110 (Delamuta et al., 2013). Random numbers were used to select farmers that were assigned to test one of the two inoculants so that each farmer tested only one. Each inoculant was tested alone, with acidulated and granulated Minjingu hyper phosphate (0–30–0 + 38CaO) or Sympal (0:23:15 + 10CaO + 4S + 1MgO + 0.1Zn) in full factorial multi-locational trials. Phosphorus rate of 30 kg P ha^−1^ was used. On each farm only one replicate was used; hence 6 plots were installed on each farm. No inputs were applied in the absolute control plots.

The plot sizes were 4.5 × 5 m and the treatments were completely randomized on each farm. Sweet potato was planted at 1 m inter-plot spacing to act as a buffer to prevent inter-plot contamination. Inoculation with Legumefix or Biofix was done at planting as a seed coating using the directions for use in the respective product labels. Each plot had 6 soybean lines of 5 m in length with 5 cm plant to plant within-row spacing and 50 cm between rows. Inoculation was done on all the rows. Soybean TGx1740-2F with medium maturity (95–100 days) (Kueneman et al., 1984) was used as the test crop. The experiment was repeated for three seasons; the location of plots and treatment allocations were kept unchanged.

### 2.3 Nodulation assessment

This was done at 50% podding when nodules are expected to be fully functional. In one of the inner inoculated rows about 50 cm from the beginning of the line, a length of 50 cm was cut and the roots and nodules were dug out. Data on the number of plants and the nodule biomass were taken. The nodules were surface sterilized and stored in glycerol (Thuita et al., 2012) for determination of nodule occupancy. Nodulation assessment was done for each of the three seasons for all treatments.

### 2.4 Harvesting

This was done at physiological maturity of the crop within the effective area, omitting the outer lines and the sampled row. Weights were taken at harvest and after oven drying to determine grain yields

### 2.5 Nodule occupancy

This was done for nodules obtained during biomass sampling at mid-podding. Before DNA extraction, the nodules were surface sterilized using 70% ethanol for 30 s and 3.3% Ca (ClO)_2_ for 2 min, then rinsed three times with sterile distilled water. One nodule was crushed in 150 μl of sterile water and DNA was directly extracted (Thiao et al., 2004). Total genomic DNA was extracted separately from 10 nodules per treatment for 33% of the farms, i.e., a total of 10 nodules for each treatment per farm. Nodule occupancy was taken for two seasons (LR2014 and SR2014) in 12 farms selected randomly in the LR2014 season. The same farms were repeated for the short rains season. From the nodules sampled at biomass assessment stage, 10 nodules were picked randomly for each inoculant with and without fertilizer (Sympal or Minjingu) per farm.

#### 2.5.1 DNA amplification and restriction

Genetic diversity was determined by Polymerase Chain Reaction-Restriction Fragment Length Polymorphism (PCR-RFLP) amplification and restriction of the 16S-23S rDNA intergenic spacer region. A 930–1100 bp intergenic region between the 16S and 23S rDNA was amplified by PCR using rhizobia-specific primers derived from the 3′ end of the 16S rDNA (FGPS 1490-72; 5′-TGCGGCTGGATCCCCTCCTT-3′) (Navarro et al., 1992) and from the 5′ end of the 23S rDNA (FGPL 132-38; 5′-CCGGGTTTCCCCATTCGG-3′) (Ponsonnet and Nesme, 1994). The PCR amplification was carried out in a 25 μl reaction volume containing 2 μl of total DNA extract, 10 pmol of each primer, and one freeze-dried bead (puReTaq Ready-To-Go PCR beads, GE Healthcare UK Ltd) containing 2.5 U of Taq DNA polymerase, 200 μM in 10 mM Tris-HCl (pH 9 at room temperature) of each dNTP, 50 mM KCl, and 1.5 mM MgCl_2_. The PCR amplification was performed in a Bio-Rad iCycler™ thermal cycler adjusted to the following program: initial denaturation for 5 min at 94 °C, 35 cycles of denaturation (30 s at 94 °C), annealing (30 s at 58 °C) and extension (30 s at 72 °C) and a final extension (7 min at 72 °C).

The PCR products were visualized by electrophoresis of 3 μl of the amplified DNA on 2% horizontal agarose gel in TBE buffer (1.1% Tris-HCl, 0.1% Na_2_EDTA·2H_2_O, and 0.55% boric acid), pre-stained with 0.033 mg ml^−1^ of Ethidium Bromide. The gel was photographed under UV illumination with Gel Doc (BIO-RAD) Software (USA). Aliquots (10 μl) of PCR products were digested with the restriction endonucleases *Msp*I and *Hae*III (5 U) in a total volume of 15 μl for 2 h at 37 °C. The restriction fragments were separated by horizontal electrophoresis in 1X TBE buffer with 3% agarose gel prestained with 0.033 mg ml^−1^ of Ethidium Bromide. The gels were run at 100 V for 3 h and photographed under UV illumination with Gel Doc (BIO-RAD, USA) software. Strains with identical restriction fragment profiles (in individual fragment size and number) were classified into the same intergenic spacer (IGS) group.

### 2.6 Characterization of study soils

Soils were collected at a depth of 0–20 cm, air dried, and thoroughly mixed to pass through a 2 mm sieve. Sub-samples were analyzed for physical, chemical, and microbiological properties prior to planting. The soil parameters analyzed were organic Carbon determined by chromic acid digestion and spectrophotometric analysis (Heanes, 1984), total N (%) determined from a wet acid digest (Buondonno et al., 1995) and N analyzed by colorimetric analysis (Anderson and Ingram, 1993). Soil texture was determined using the hydrometer method; soil pH in water was determined in a 1:2.5 (w/v) soil: water suspension and available P using the Mehlich-3 procedure (Mehlich, 1984). The resulting extracts were analyzed using the molybdate blue procedure described by Murphy and Riley (1962). Exchangeable cations (Ca, Mg, and K) were extracted using the Mehlich-3 procedure and determined by atomic absorption spectrophotometry. A fresh soil sample was used in the estimation of rhizobia in the soils using the most probable number count as described by Brockwell et al. (1995). Soybean variety TGx1740-2F was used as a trap crop grown in N-free and autoclaved sterile sand.

### 2.7 Data analysis

All the data of grain yield and nodulation were subjected to Analysis of Variance to evaluate the effect and interaction between inoculant and P source on grain yield and nodule fresh weight using the mixed procedures of the SAS System (SAS Institute Inc, 2006). The effects of the different treatments were compared by computing least square means and means were separated using least significant differences (LSD); the significance of difference was evaluated at *P <* 0.05. In the mixed model analysis, ‘farmer’ within ‘village’ were considered as random factors and nested within ‘village’ (Pypers et al., 2011).

The value cost ratio (VCR) was computed using Eq. (1).

(1) $$VCR=\frac{Unit\;price\;of\;the\;produce\;\left(\$\;kg^{-1}\right)\times Yield\;gain\;\left(kg\;ha^{-1}\right)}{Cost\;of\;the\;inputs\;\left(\$\;ha^{-1}\right)}$$

The values obtained were then used to draw frequency cumulative curves with grain yield increases and a VCR value of 3 used to determine profitability (Thompson, 1991).

## 3 Results and discussion

### 3.1 Initial soils

The summary of soil characterization data is shown in Table 1. About 50% of the soils (59) had pH lower than 5.5 which would not be considered ideal for soybean cultivation. These pH levels are common in the western Kenya region dominated by Ferralsols and Acrisols with a high rainfall. The other soils had a pH range of 5.5–6.5. Soil available P was categorized as low to very low (less than 25 mg P kg^−1^ of soil) for 87% of the soils and thus it would be necessary for farmers to include a P source for soybean production. Only 8 farms had P in levels that could be considered adequate for the short term. About 60% of the soils had low to very low N (less than 0.12% N). Other soils had moderate N (highest 0.2% N) and therefore a response to inoculation was expected as soil N would not be adequate to support optimal soybean growth.

**Table 1:** Soil analysis and ratings of the levels of selected parameters for the 107 soil samples from the trial sites.

Parameter	Mean	Minimum	Maximum	Standard deviation	CV (%)	Very low	Low	Moderate	High	Very high
P (mg kg^−1^)	11	1	161	2.360	35	89	4	3	3	8
Total N (%)	0.11	0.04	0.20	0.003	30	3	68	46	0	0
Organic C (%)	1.46	0.65	2.35	0.041	29	0	60	47	0	0
Ca (mg kg^−1^)	430	74	1151	23.06	56	72	31	4	0	0
Mg (mg kg^−1^)	166	28	664	9.279	58	0	3	11	56	37
K (mg kg^−1^)	44	8	189	3.118	74	69	31	6	1	0
pH	5.43	4.05	6.78	0.058	11	4.00–4.99	5.00–5.50	5.51–6.00	6.01–7.00	7.01>
						29	30	30	18	0

Organic Carbon was rated as either low or moderate (0.5–3% C) for all the farmers; 64% had a K rating of very low (0–120 mg K kg^−1^ of soil) and 29% as low (121–160 mg K kg^−1^ of soil). Similarly, a deficiency in calcium was common with 103 of the 107 farms having levels rated as low (9500–1000 mg Ca kg^−1^ soil) and very low (<500 mg Ca kg^−1^). The data from soil chemical analysis showed low soil pH, N, and P, and widespread deficiency in K and Ca.

The soils had variable levels of indigenous rhizobia capable of nodulating TGx1740-2F as indicated by the MPN tests (Table 1). Some soils had zero while the highest population of rhizobia was recorded as 328 CFU g^−1^ of soil. The sites were suitable for testing the effectiveness and competitiveness of the inoculants used in an environment with varying physical, chemical, and biological levels representing typical farm conditions in most smallholder farming communities. It is considered a standard practice to include a P source in most legume inoculation with rhizobia in most of SSA and worldwide owing to the widespread deficiency of P and its key role in BNF (Vance et al., 2000). However, this study shows that deficiency of K is common in most of the farms even though P deficiency remains prevalent, This could be attributed to the fact that fertilizers, even where they are used, have formulations that are devoid of K and mainly contain N and P. This has led to a continuous removal without replenishment. Biological N fixation is particularly sensitive to environmental stress such as nutrient deficiency (Divito and Sadras, 2014).

### 3.2 Soybean nodulation

**Fig. 1:**
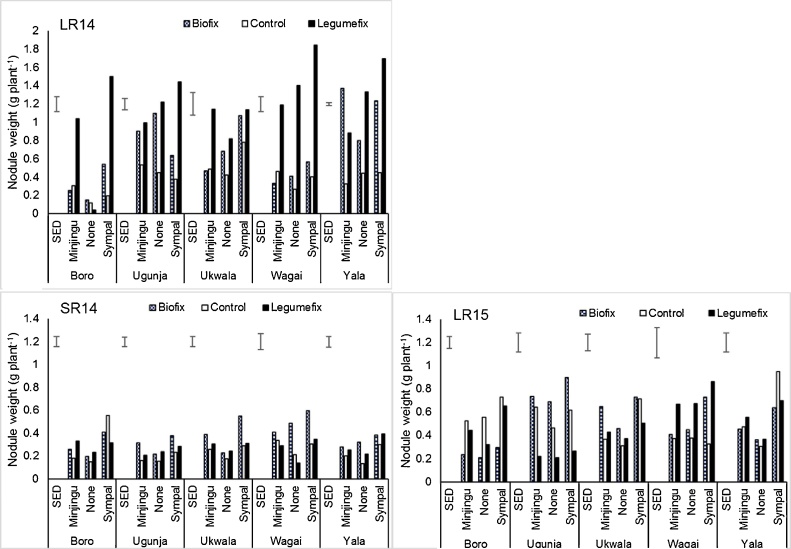
Mean fresh weight of soybean nodules for the 107 farms distributed in five Divisions during the three cropping seasons in the multi-locational trial in Siaya County as affected by the treatments. Error bars represents the Standard error of the difference (SED). LR14-long rains 2014, SR14-Short rains 2014, LR15-Long rains 2015.

Fig. 1 shows soybean nodulation for the three cropping seasons as determined at mid-podding with significant differences (*P <* 0.05) due to inoculation, P source, and their interaction. The highest nodulation was observed in season 1 of LR2014 for all the treatments and a significant reduction was observed in the next two seasons (SR2014 and LR2015). In each season the non-inoculated controls (Control – None) had the lowest nodulation that was attributed to the low initial population of rhizobia in most soils as indicated by the MPN tests. As expected no differences were observed between the control group using Biofix and those with Legumefix. Similarly, there were no significant differences (*P <* 0.05) between Minjingu for farmers using Biofix and those using Legumefix.

The highest nodulation was observed when the inoculants were combined with a P source (Fig. 1). Legumefix + Sympal gave the highest nodulation (1.5 g of nodules plant^−1^) in Wagai Division. There were no significant differences between non-inoculated controls and those receiving either Minjingu or Sympal alone in all the divisions, indicating that inoculation is paramount for increased nodulation and BNF to be observed. It was also noted that there was higher nodulation where Sympal + inoculant was used compared to Minjingu + inoculant and this was probably due to the difference in the formulation of the two products. The trend in nodulation for the three seasons was similar with the lowest being in Controls, slightly higher (even though not significant) in plots receiving P, and highest in P + inoculant plots. Nodulation was highest in season 1 (LR2014) and lower in both SR2014 and LR2015.

Application of Sympal (Containing 0:23:15[N: P2O5:K2O] + 10CaO + 4S + 1MgO + 0.1Zn) led to a response for both nodulation and grain yields compared to Minjingu (containing P and Ca). This is in agreement with the findings that reported K and S are among the important drivers of BNF (Atieno et al., 2012; Mia et al., 2014). Nodulation increased with inoculation even though variety TGx1740-2F is promiscuous and this is in agreement with previous findings (Atieno et al., 2012; Thuita et al., 2012; Ronner et al., 2016). Nodulation was highest in season 1 compared to seasons 2 and 3; this could be due in part to residual N in the soil. Nitrogen levels in soils have been shown to affect nodulation (Gibson and Harper, 1985) and benefit the next crop. Nodule mass has been shown to be more sensitive to nutrient deficiency than shoot mass; this may explain improved nodulation when Legumefix and Sympal were combined than when used singly, indicating a greater need for nutrition to maintain rapid growth and functioning of nodules.

### 3.3 Nodule occupancy

Nodule occupancy was determined for 33% of the inoculated soybean plots for the LR2014 and SR2014 cropping seasons (Table 2). Inoculation in the LR2014 season was considered successful except for Legumefix alone that did not meet the 66% nodule occupancy rate established as critical for effective inoculation by the introduced strain (Thies et al., 1991). Nodule occupancy rates were higher in Biofix (strain USDA110) than in Legumefix (strain 532c) suggesting that strain USDA110 could be more competitive than strain 532c under the soil conditions in which the two strains were tested. During the SR2014 cropping season, nodule occupancy rates for Biofix were reduced compared to the LR2014 season but remained above the critical point except for Biofix alone which was 63%. However, nodule occupancy rates for Legumefix increased above 66%, and this could be attributed to the persistence of rhizobia from the long rains season. This could have given strain 532c a competitive advantage (through numbers) as the nodulation rates were much higher than in Biofix in the LR2014 cropping season (Fig. 1). Phosphorus source did not significantly (*P <* 0.05) affect nodule occupancy but having a P source was important especially for strain 532c which gave better nodule occupancy with P than without it. This could be related to the N-fixing efficiency of the strains. The more efficient strain 532c is likely to be more sensitive than the strain USDA110 to an adequate P supply for the energy-demanding process of BNF.

**Table 2:** Nodule occupancy for the inoculated soybean during long and short rains 2014 cropping seasons.

Treatment	LR2014 season		SR2014 season	
	Nodule No.	Occupancy (%)	Nodule No.	Occupancy (%)
Biofix	120	88	120	63
Biofix + Minjingu	120	85	120	83
Biofix + Sympal	120	91	120	79
Legumefix	120	57	120	68
Legumefix + Minjingu	120	67	120	77
Legumefix + Sympal	120	67	120	70

The nodules are from 12 farms sampled in both seasons.

Nodule occupancy results indicated that strain USDA110 (Biofix) had a high nodule occupancy rate regardless of P application, suggesting that it could be more adapted to the local environmental condition. The strain USDA100 in Biofix could be less sensitive to the nutrient deficiency-related stress. This may explain the widespread use of USDA110 in inoculum production worldwide and could have made it more competitive than strain 532c under the conditions of the study. The increased nodule occupancy rates in season 2 for Legumefix may seem to support this theory as the residual effect of the season 1 crop would have slightly improved the soil nutrient status as it was also accompanied by a reduction in overall nodule mass. It was also considered a successful inoculation campaign as the 66% threshold of nodule occupancy was met (Thies et al., 1991).

Increased nodulation and high levels of nodule occupancy by the introduced strains resulted in increased grain yields in each of the three seasons. The residual benefits from season 1 were seen with grain yields increasing every season. This has been reported previously on subsequent crops (legumes) and non-legumes in mixed stands (Graham and Vance, 2000). Addressing nutrient limitations other than P has allowed increases beyond any previously reported levels of 2000 kg ha^−1^ (Mpepereki et al., 2000; Musiyiwa et al., 2005; Ronner et al., 2016) and compared favorably with the best fields achieving yields of 3500–4000 kg ha^−1^ that are similar to results from Brazil and Argentina (Hungria et al., 2006; Melchiorre et al., 2011). This may not only increase profitability but also improve soil fertility and make inoculation in the context of ISFM attractive for farmers, as well as for sustainable agricultural intensification.

### 3.4 Grain yields

**Fig. 2:**
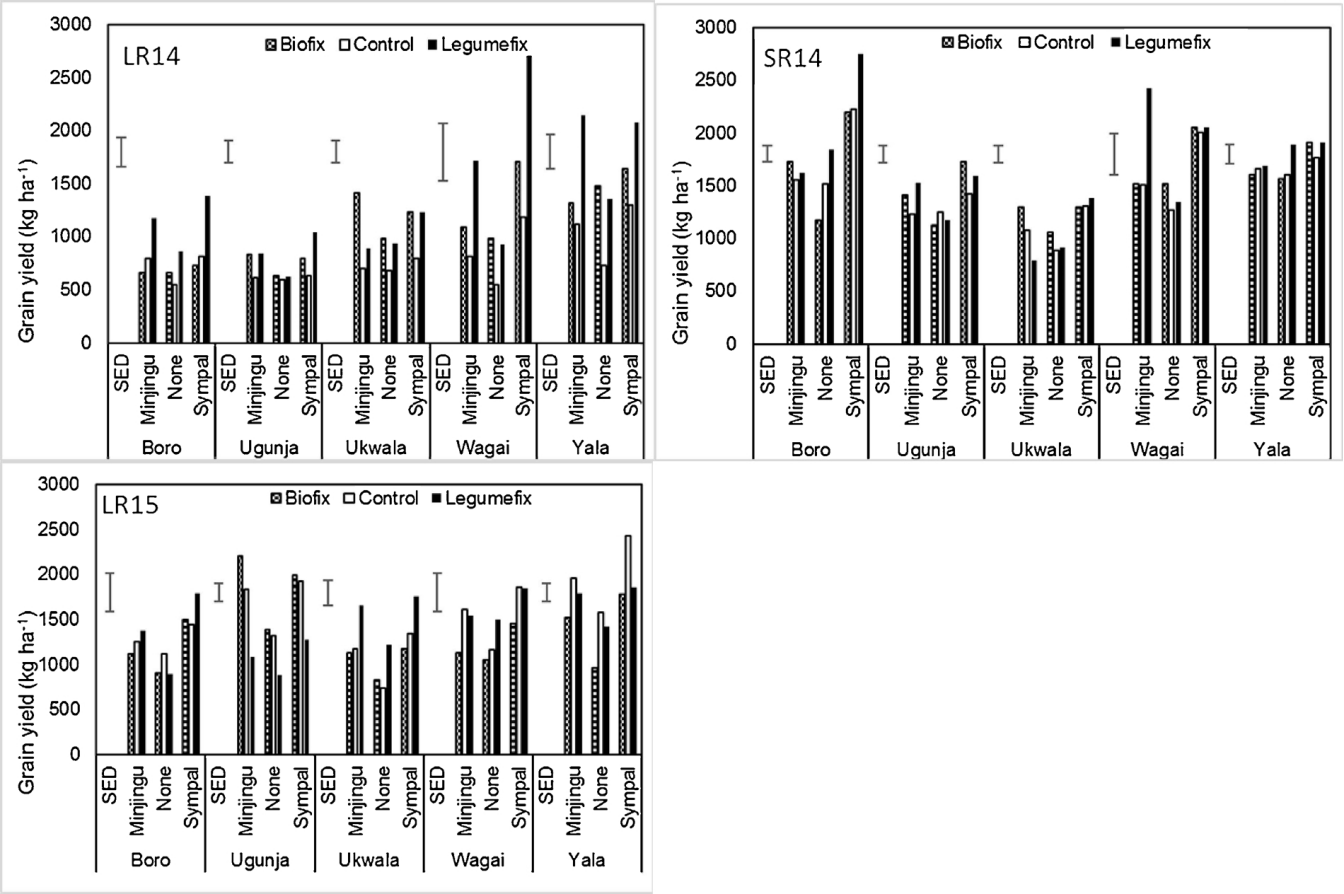
Mean soybean grain yields in the five divisions during the three cropping seasons in the multi-locational trial in Siaya County as affected by the treatments. Error bars represents the Standard error of the difference (SED). LR14-long rains 2014, SR14-Short rains 2014, LR15-Long rains 2015.

There were significant differences in grain yields due to treatments in all three cropping seasons (Fig. 2). The interaction of inoculation and P source resulted in increased grain yields for the three seasons but yields were higher for seasons 2 and 3 compared to season 1 (Fig. 2). On average the lowest yields were obtained from the absolute control treatments (No P and no inoculation) in the LR2014 season with control farmers getting a mean yield of less than 700 kg ha^−1^. Application of Minjingu or Sympal led to a significant yield gain (*P <* 0.05) above the controls of about 400 kg ha^−1^. Similar yield increases were obtained when inoculations with Biofix or Legumefix were done, suggesting that P or inoculation alone would lead to only modest yield increases. When inoculant and P source were combined yield increases were about 1000 kg ha^−1^ above the Control. The yield trends for the three seasons remained the same as in the LR2014 but were significantly higher for all treatments except the non-treated controls and Biofix alone. Table 3 shows a summary of the yield variability during the three cropping seasons. The responses to the treatment varied with very low yields (less than 350 kg ha^−1^) in some farms in each of the treatments and much higher yields that were more than double the treatment mean observed in all the treatments except for the control, Minjingu + Biofix, and Sympal alone (Table 3). This wide variation was related to the soil fertility gradients with the very poor soils giving low yields and those with a better level of fertility giving higher yields in the control.

**Table 3:** Selected descriptive statistics for soybean grain yields from the three seasons indicating the extent of variability in yield as affected by the treatments in all five divisions.

Parameter	Biofix	Minjingu + Biofix	Sympal + Biofix	Control	Minjingu + Legumefix	Sympal + Legumefix	Legumefix	Minjingu	Sympal
	1128	1338	1619	923	1650	1984	1351	1179	1367
Median	1048	1271	1571	868	1569	2056	1295	1102	1353
Standard Deviation	746	786	802	553	853	907	731	650	688
CV %	66	59	50	60	52	46	54	55	50
Minimum	42	59	164	9	128	311	139	102	36
Maximum	3042	3517	3707	2869	3913	4260	3187	3342	3073

The values are based on the three seasons.

There were significant grain yield differences (*P <* 0.05) between the five divisions with the highest mean grain yields obtained in Boro Division and the lowest in Ukwala Division (Fig. 2). Responses to treatments were low with all the treatments having grain yields lower than 1500 kg ha^−1^. By comparison in Boro Division, only Control, Minjingu, and Biofix had grain yields lower than 1500 kg ha^−1^. In each of the five divisions, Legumefix + Sympal had the highest grain yields with an average grain yield of at least 2000 kg ha^−1^ in Boro, Wagai, and Yala Divisions. The Control had the lowest grain yields in all the divisions but these were not significantly different from Biofix and Legumefix alone (Ugunja and Ukwala Divisions) and all treatments receiving Minjingu in Ukwala Division. Variability in responses to ISFM packages has been reported (Foli, 2012; Vanlauwe et al., 2014) and often relate to multiple nutrient deficiencies as well as other factors including low soil pH, hardpan development, and very low levels of soil organic matter.

**Fig. 3:**
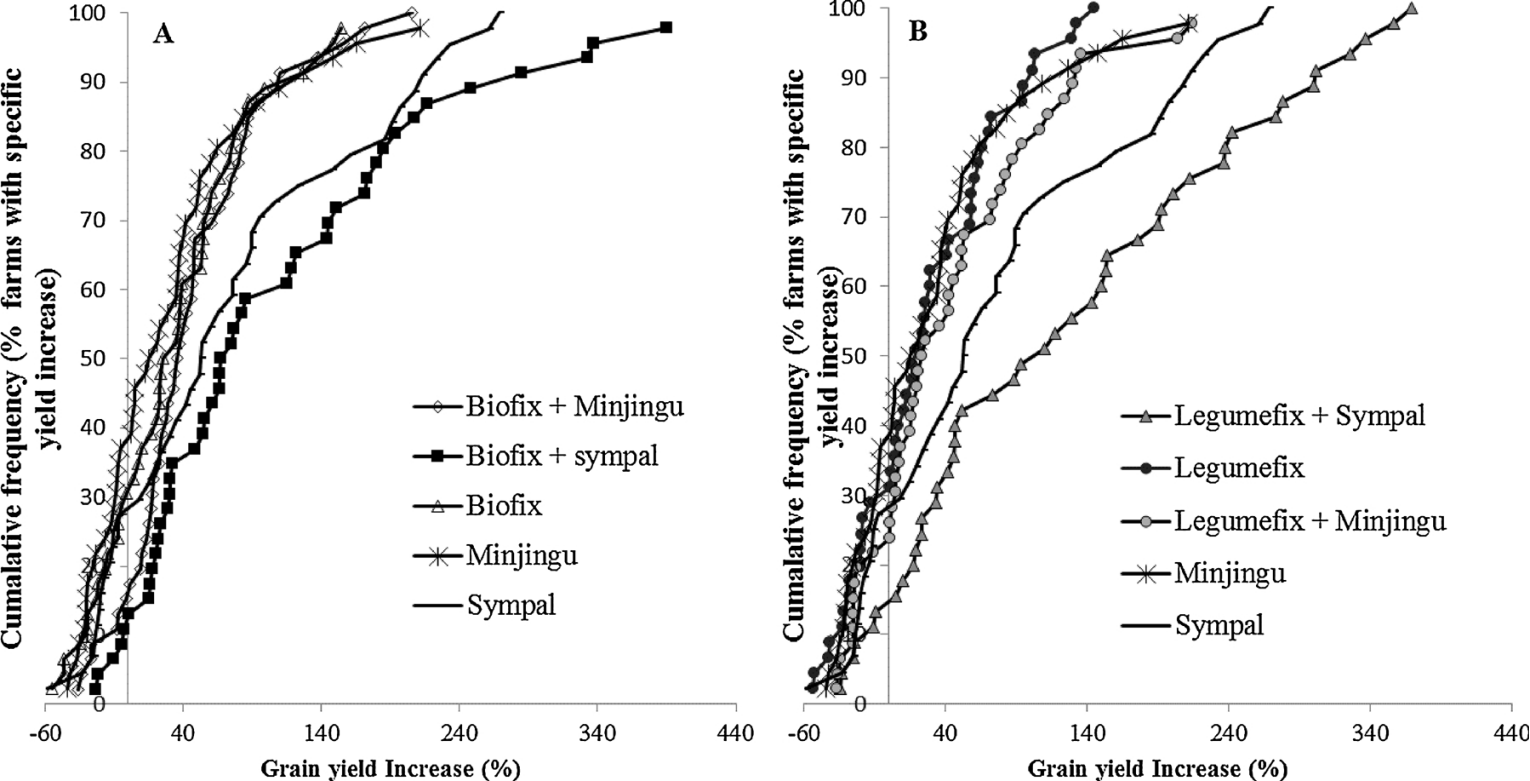
A and B: Cumulative frequency of grain yield increase against grain yield increases above the control treatment for the farmers who tested Biofix (3A) and Legumefix (3B) during Short rains 2014 cropping season in the multi-locational trial in Siaya County as affected by the treatments.

To understand the level of yield increase above the control and frequency for the SR2014 cropping season, grain yield gain (%) based on the negative control were determined and presented against the cumulative frequency of yield increase for Biofix (Fig. 3A) and Legumefix (Fig. 3B). The yield increase could be split into two groups for both Fig. 3A and B: the treatments that received Sympal with or without inoculation. The combinations of inoculant + Sympal gave the highest grain yield increases and was leaning most to the right and predicted that about 85% of the farmers would get a positive yield increase when it was used. Sympal alone was the next best with 72% chance of net yield increases being observed. All the Minjingu treatments and inoculants alone formed the group with higher chances of a low and negative yield increase (30–40%). The response to Legumefix + Minjingu had fewer chances of negative yield increases (18%) but in general the yield increases were not high, suggesting that some soils were not very suitable for Minjingu or the prevalence of other limiting nutrients meant the soybean crop would have been affected and thus the low yields. However, it reduced chances of negative yield increases in soils that had low soil pH but where nutrients were less limiting. For the most responsive farms, the yield increase was very high (100–300%) with some farms (data not shown) giving yields of 3000–4000 kg ha^−1^ grain.

Although impressive yields were observed, yields did not improve in some farms, suggesting that there could be other limiting factors that were not addressed. Minjingu hyper phosphate had better results in soils with lower soil pH and reduced incidences of the no-yield response but the yield gains remained low, probably because of limitations from other missing nutrients essential for optimal yields. Similarly, other key nutrients such as S, Mg, Ca, etc. in the context of full ISFM, may need to be investigated as we seek to improve soybean yields in the wider SSA region. Similarly, varied yields have been reported in Nigeria (Ronner et al., 2016) in a multi-locational trial encountering various levels of soil fertility constraints and suggest not only the need to investigate limiting nutrients but also to develop specific fertilizer blends to meet such limitations. The lack of a clear trend of low yields and a clear link to individual nutrient deficiency in the study soils suggested that the causes of low responses could be linked to multiple factors. This is also inherently difficult to predict as the soils used did not have the same texture and amounts of soil available N, for example, or of organic matter that can be considered low in a clay soil but high in a sandy soil.

For adoption of ISFM packages, farmers will often consider both short and long-term benefits of their returns on investment. Traditionally farmers in SSA do not use fertilizers on legumes and even where they do so, levels used are generally much lower than those recommended (Chianu et al., 2011). The low cost of inoculants, therefore, make it an attractive input for farmers but to fully benefit from inoculation, the use of inoculants in ISFM packages is recommended.

### 3.5 Value cost ratios of the various treatments

**Fig. 4:**
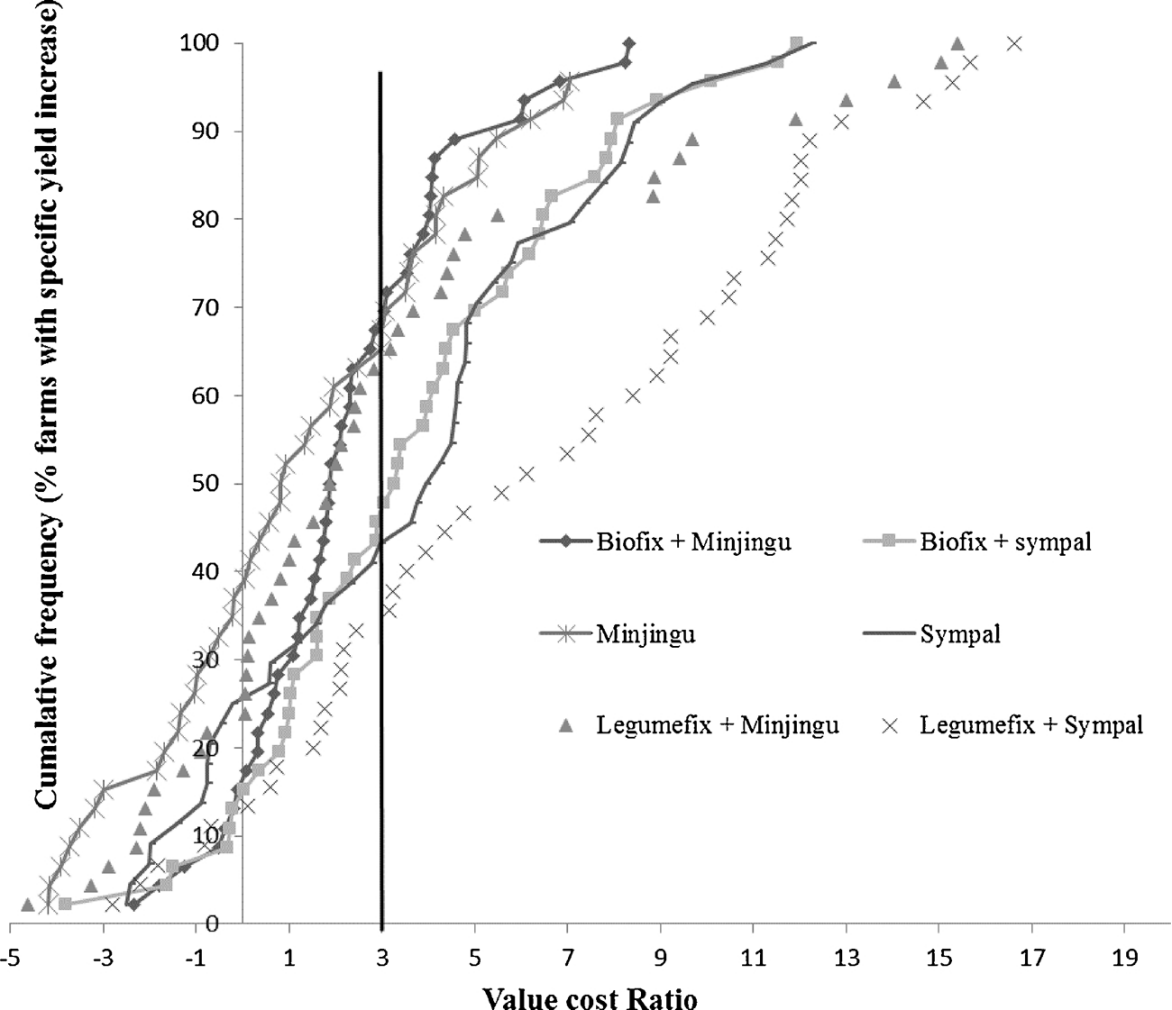
Cumulative frequency grain yield increase against Value cost ratio of the treatments for farmers who tested Legumefix and Biofix during short rains 2014 cropping season in the multi-locational trial in Siaya County as affected by the treatments. A Value cost of ≥3 was considered as profitable.

The value cost ratios (VCR) were variable across treatments (Fig. 4). Cumulative frequency curves of yield increases against VCR were drawn using a threshold value of VCR ≥ 3 (Thompson, 1991). Both inoculants alone (Legumefix and Biofix) were left out owing to the low costs of inoculants making them skew the graph as well as being a non-sustainable practice. However, they can be used initially as the farmer builds capital to invest on other nutrients. Treatments containing Sympal (Sympal alone, Sympal + Biofix, and Sympal + Legumefix) had high VCR values and therefore lower yield increases were required for profitability (35–45%) compared to Minjingu (Minjingu alone Minjingu + Biofix, and Minjingu + Legumefix) which required a yield increase of 65–70% to be profitable (Fig. 4). This suggested that Sympal was a more suitable ISFM component in the trial sites than Minjingu for the majority of the farms. However it was also noted that there were farms in which none of the ISFM packages resulted in yield increases and thus would require additional study to be understood.

The yield responses and profitability of the inoculation and addition of either Sympal or Minjingu also depended on soil conditions in individual farms. Although the use of inoculants was economically very attractive due to their low prices this is only as a starter in a stepwise introduction of inoculants and its accompanying ISFM packages for smallholder farmers to avoid further mining of soil nutrients that would lead to a decline in yields (Ronner et al., 2016). In the long term, addition of inorganic nutrients would be reduced hence increasing the profitability of soybean production owing to the build-up of residual nutrients that would both increase yield and reduce input costs.

## 4 Conclusion

On average, yields of the TGx1740-2F variety of soybean were relatively low in Siaya County of western Kenya (2000 kg ha^−1^). Soil analysis showed deficiencies in most of the nutrients tested (e.g., N, P, K, and Ca). Thus, it was possible to raise soybean grain yields up to 4000 kg ha^−1^ through using rhizobia inoculation and by addressing selected nutrient deficiencies. For a sustainable economic return from using rhizobia inoculants in soybean production in Siaya County of western Kenya, we recommend that their use in the context of ISFM considers other potential limiting factors such as secondary and micro-nutrients, as well as soil organic matter and pH. The geographical spread of the study region which has three agroecological zones demonstrated the need for ISFM packages that can respond to limitations in soil fertility for increased nodulation and grain yields.

## Funding

This work was supported by IITA through the COMPRO-II project funded by the Bill and Melinda Gates Foundation (USA) and the RCT study implemented by the Paris School of Economics and funded by the *Conseil National de Recherche Scientifique* (France).
